# Sciatic nerve repair using poly(ε‐caprolactone) tubular prosthesis associated with nanoparticles of carbon and graphene

**DOI:** 10.1002/brb3.755

**Published:** 2017-06-30

**Authors:** Kyl Assaf, Claudenete Vieira Leal, Mariana Silveira Derami, Eliana Aparecida de Rezende Duek, Helder Jose Ceragioli, Alexandre Leite Rodrigues de Oliveira

**Affiliations:** ^1^ Department of Structural and Functional Biology Institute of Biology University of Campinas – UNICAMP Campinas Brazil; ^2^ Department of Materials Engineering Faculty of Mechanical Engineering University of Campinas – UNICAMP Campinas Brazil; ^3^ Department of Physiological Sciences Biomaterials Laboratory PUC‐SP Brazil; ^4^ Faculty of Electric Engineering and Computation (FEEC) University of Campinas – UNICAMP Campinas Brazil

**Keywords:** carbon nanotubes, graphene, PCL, peripheral nerves, tubulization

## Abstract

**Introduction:**

Injuries to peripheral nerves generate disconnection between spinal neurons and the target organ. Due to retraction of the nerve stumps, end‐to‐end neurorrhaphy is usually unfeasible. In such cases, autologous grafts are widely used, nonetheless with some disadvantages, such as mismatching of donor nerve dimensions and formation of painful neuromas at the donor area. Tubulization, using bioresorbable polymers, can potentially replace nerve grafting, although improvements are still necessary. Among promising bioresorbable synthetic polymers, poly(l‐lactic acid) (PLLA) and poly(ε‐caprolactone) (PCL) are the most studied. Carbon nanotubes and graphene sheets have been proposed, however, as adjuvants to improve mechanical and regenerative properties of tubular prostheses. Thus, the present work evaluated nerve tubulization repair following association of PCL with nanoparticles of carbon (NPC) and graphene (NPG).

**Methods:**

For that, adult Lewis rats were subjected to unilateral sciatic nerve tubulization and allowed to survive for up to 8 and 12 weeks postsurgery.

**Results:**

Nanocomposites mechanical/chemical evaluation showed that nanoparticles do not alter PCL crystallinity, yet providing reinforcement of polymer matrix. Thus, there was a decrease in the enthalpy of melting when the mixture of PCL + NPC + NPG was used. Nanocomposites displayed positive changes in molecular mobility in the amorphous phase of the polymer. Also, the loss modulus (E”) and the glass transition exhibited highest values for PCL + NPC + NPG. Scanning electron microscopy analysis revealed that PCL + NPC + NPG prostheses showed improved cell adhesion as compared to PCL alone. Surgical procedures with PCL + NPC + NPG were facilitated due to improved flexibility of the prosthesis, resulting in better stump positioning accuracy. In turn, a twofold increased number of myelinated axons was found in such repaired nerves. Consistent with that, target muscle atrophy protection has been observed.

**Conclusion:**

Overall, the present data show that nanocomposite PCL tubes facilitate nerve repair and result in a better regenerative outcome, what may, in turn, represent a new alternative to pure PCL or PLLA prostheses.

## 1 INTRODUCTION

The peripheral nervous system has the remarkable capacity of regenerating following traumatic injury (Cunha, Panseri, & Antonini, 2011; Lewis, Courchet, & Polleux, 2013; Lopes et al., 2006). When there are significant gaps created by retraction or by extensive nerve damage resulting in loss of a nerve segment, grafts are required (Moroder et al., 2011). However, nerve autografts have some disadvantages, such as availability of donor tissue, the formation of painful neuromas in the donor area, and loss of donor nerve function (Bockelmann et al., 2011; Evans, 2000; Moroder et al., 2011; Nakamura et al., 2004; Pierucci, de Duek, & de Oliveira, 2008).

In the medical practice, tubular silicone prostheses are already used to bridge gaps between severed nerve stumps (Dahlin, Anagnostaki, & Lundborg, 2001; Dahlin & Lundborg, 2001; Lundborg, Dahlin, & Danielsen, 1991; Lundborg, Rosen, Abrahamson, Dahlin, & Danielsen, 1994; Lundborg, Rosen, Dahlin, Danielsen, & Holmberg, 1997; Lundborg, Rosen, Dahlin, Holmberg, & Rosen, 2004). Although the recipient well accepts such prostheses, they are not degradable and require a second surgery for their removal (Cunha et al., 2011). In this sense, many aspects of the tube used for nerve regeneration must be considered, for example, if it is bioresorbable, meaning that it undergoes degradation and the products and subproducts of the process are removed by metabolic pathways, causing no side effects. Additionally, there are also other important factors such as the degradation time frame, flexibility, and porosity of the material (Brushart, Mathur, Sood, & Koschorke, 1995; Evans, 2000; Moore et al., 2009; Rezwan, Chen, Blaker, & Boccaccini, 2006; Sun & Downes, 2009).

The most often utilized materials are poly(α‐hydroxy acids), a very promising bioresorbable synthetic polymer family. The chemical properties of these polymers allow hydrolytic degradation through de‐esterification and its monomeric components are removed by metabolic pathways (Rezwan et al., 2006). The most studied bioresorbable polymers are poly(glycolic acid) (PGA), poly(para‐dioxanone) (PPD), poly(l‐lactic acid) (PLLA), and poly(ε‐caprolactone) (PCL) (Rezwan et al., 2006; Sun & Downes, 2009).

Poly(ε‐caprolactone) is a biomaterial that provides many of the desired properties for a tubing used for nerve regeneration. It is easy to handle and gives transparency, allowing better visualization of nerve stumps during surgery. It also provides flexibility, facilitating suturing of nerve stumps to the tubular prosthesis wall (Pierucci et al., 2008). Furthermore, it provides an excellent substrate for guided cell migration and axonal growth, being an efficient alternative to peripheral nerve autografting (Bockelmann et al., 2011; Pierucci et al., 2008; Sun & Downes, 2009; Sun et al., 2010; Yu et al., 2011).

Nanometric supports have also been implemented to promote growth and development of neurons and non‐neural cells (Mattson, Haddon, & Rao, 2000). The large class of nanomaterials currently available contains many families of nanostructures (Kotov et al., 2009). Carbon nanostructures are the most evident products of nanotechnology and, among them, there are carbon nanotubes, carbon nanofibers, graphene, and a variety of carbon forms (Armentano, Dottori, Fortunati, Mattioli, & Kenny, 2010).

A carbon nanotube is characterized by rolling one or more graphene sheets concentrically with a diameter of nanometric dimensions and hollow inner cavity (de Oliveira, Ceragioli, & Assaf, 2014). Carbon nanotubes are considered key materials and have many possibilities for technological applications due to properties such as high chemical resistance, oxidation and temperature resistance, low density, electrical conductivity, mechanical strength, and flexibility (de Oliveira et al., 2014). All these features make them excellent devices for neural implants, helping structurally and functionally for regeneration of damaged axons (Kotov et al., 2009; Sun, Sun, Li, & Peng, 2013). Carbon nanotubes can also be excellent candidates for improving the properties of polymer composites due to features such as longitudinal elasticity, thermal and electrical conductivity, and low density (Terrones, 2013). When added to a polymer, they offer improvements in mechanical strength (Armentano et al., 2010). Liao et al. (2012) found that fibrous membranes of PLLA/PCL have benefits when added carbon nanotubes, such as increased mechanical properties, improved biodegradation, cell proliferation, and reorientation.

Graphene is also considered a promising material. Polymer nanocomposites filled with graphene have shown significant improvements in their properties such as tensile strength, electrical conductivity, and thermal stability (Zhu et al., 2010). In biomedical applications, graphene offers advantages to the nervous system, since the neural cells are electroactive and the electronic properties of graphene can be adapted for cargo transport required for cell electrical interface (Kotov et al., 2009). Furthermore, chemical stability of graphene facilitates integration with neural tissues (Kotov et al., 2009). Li et al. (2011) observed that graphene films exhibit excellent biocompatibility in primary cultured rat hippocampal neurons and are also capable of promoting growth and sprouting of neurons, especially during the early development phase.

Therefore, the purpose of this study is to assess peripheral nerve regeneration, through transection of the sciatic nerve in rats, using tubular prostheses made of PCL alone or associated with nanoparticles of carbon (NPC) and graphene (NPG). Chemical and mechanical properties of the nanocomposites are also evaluated, demonstrating advantages of such associations.

## 2 MATERIALS AND METHODS

### 2.1 Construction of the prosthesis for tubulization

All biomaterial/composite membranes used to obtain tubular prostheses were prepared by a solvent evaporation technique as described previously (Pierucci et al., 2008). First, PCL was dissolved in chloroform (Merck, Germany) and kept under stirring for 2 hr for complete dissolution of the polymer. At the same time, solutions of carbon nanostructures were prepared in concentrations of 0.5% of NPC (multiwall), 0.5% of graphene oxide—NPG and 0.5% of the mixture of nanostructures (0.25% of NPC plus 0.25% of NPG) in chloroform. Such solutions were placed in the ultrasonic bath for dispersion. NPC were prepared by HFCVD (Hot Filament Chemical Vapor Deposition) using acetone as carbon atoms source on a polished copper foil substrate as described previously (Grecco et al., 2011). The processing for NPG was the same described before to maintain identical properties (Mendonca et al., 2015). Briefly, NPG were prepared after catalytic conversion using a copper substratum to which 1 ml of polyaniline diluted in dimethylformamide (Synth, São Paulo, SP, Brazil) was added. After drying for 2 hr at room temperature, 0.2 ml of nickel nitrate dissolved in pure acetone (Synth) was added to the preparation which was subsequently placed within a chemical vapor deposition reactor assisted by a hot filament. The hydrocarbons used as a carbon source were camphor and acetone.

The dispersed solutions of carbon nanostructures were mixed with the PCL solution and homogenized. These composites and PCL‐alone solution were placed on glass molds into a glass vat for evaporation of the solvent and obtainment of the membranes.

The membranes were then rolled onto 1.6 mm diameter pins (Evans et al., 1999), sealed with surgical glue (Histoacryl, n‐butyl‐2‐cyanoacrylate, Braun, Germany), and cut into 10‐mm length prostheses.

### 2.2 Characterization of nanocomposites

#### 2.2.1 Fracture surfaces of the membranes

To evaluate the morphology, the fracture surfaces of pure PCL and nanocomposites were analyzed in a scanning electron microscope (Zeiss EVO MA15) at 10 kV. Fractures of the membranes were obtained after freezing in liquid nitrogen. The samples were metalized in a sputter coater (BAL‐TEC SCD050) with gold.

#### 2.2.2 Differential scanning calorimetry

The differential scanning calorimetry (DSC) was performed on Netzsch equipment (DSC 200 F3 Maia, Germany). The samples were heated at 25–200°C, maintained for 5 min, after cooled down to −100°C, held for 5 min, and then heated at 100°C. Masses were used between 8 and 10 mg, heating rate of 10°C/min, a nitrogen atmosphere at 50 ml/min.

#### 2.2.3 Dynamic mechanical analysis

The dynamic mechanical analysis (DMA) was performed on Netzsch equipment (DMA 242). Samples were assessed at −100 to 60°C at a rate of 5°C/min, on the shear stress mode.

### 2.3 Experimental groups

Forty adult female Lewis rats were obtained from the Multidisciplinary Center for Biological Investigation (CEMIB/UNICAMP) and housed under a 12‐hr light/dark cycle with free access to food and water. They were divided into four groups according to the composition of the implanted prosthesis: PCL (*n* = 10), PCL containing carbon nanotubes (NPC) (*n* = 10), PCL containing graphene oxide (NPG) (*n* = 10), and PCL containing carbon nanotubes and graphene oxide (a mixture of NPC + NPG) (*n* = 10). In each group, animals were sacrificed after a postsurgery survival period of 8 and 12 weeks (*n* = 5 per survival time). All procedures were done in accordance with the ethical principles regulated by the National Council of Animal Experimentation (CONCEA) and with the approval of the Ethics Committee on Animal Experimentation of University of Campinas (CEUA/UNICAMP, protocol no. 2617‐1).

### 2.4 Tubulization

**Figure 1:**
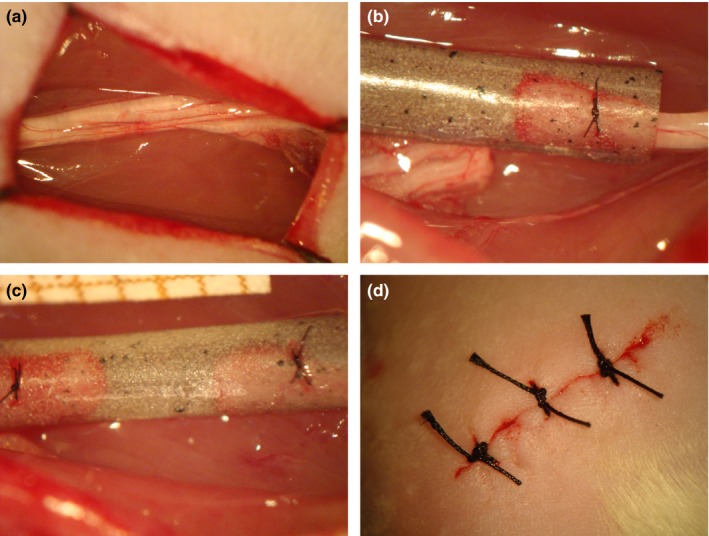
Tubulization of the sciatic nerve by using a PCL + NPC + NPG prosthesis. (a) Exposure of the left sciatic nerve. (b) Sutured distal stump to the end of the tube. (c) Sutured stumps into the tube. (d) Animal skin sutures at the incision site. Note the tube transparency, allowing correct alignment of the stumps, leaving a 3‐ to 4‐mm gap. PCL, poly(ε‐caprolactone); NPC, nanoparticles of carbon; NPG, nanoparticles of graphene

Under anesthesia, obtained with a mixture of Kensol (xylazine, Köning, 10 mg/kg) and Vetaset (Ketamin, Fort Dodge, 60 mg/kg) (1:1, 0.2 ml/100 g, i.p.), the left sciatic nerve of the animals was exposed and transected at the midthigh level (Figure 1a). In sequence, the stumps of the nerve were introduced into the tubular prosthesis and fixed to its wall with two single stitches (8‐0, mono nylon suture, Ethicon), maintaining the alignment and leaving a 3‐ to 4‐mm gap between the stumps (Figure 1b,c). Completed the tubulization procedures, the muscular layer was sutured with 7‐0 silk suture and the skin closed with three stitches (4‐0, mono nylon, Ethicon) (Figure 1d).

### 2.5 Specimen preparation

After 8 and 12 weeks postsurgery, the rats were perfused transcardially with phosphate‐buffered saline (0.9% NaCl, PB 0.1 mol/L, pH 7.4) followed by Karnovsky solution (2% glutaraldehyde, 1% paraformaldehyde, PB 0.1 mol/L, pH 7.4). The contralateral and ipsilateral nerves were carefully dissected out under a surgical microscope. The ipsilateral nerve was dissected such that the proximal and distal stumps were separated. In sequence, the specimens were postfixed with 1% osmium tetroxide for 3 hr, washed with water, dehydrated with alcohol and acetone, and processed for resin embedding (Epon 812, EMS). Transverse semi‐thin sections (0.5 μm thick) were obtained and stained with Sudan Black for morphometric analyses, counting of the regenerated fibers and measurement of the total nerve area.

Soleus and tibialis cranialis muscles were dissected out, on both sides, 12 weeks postsurgery (*n* = 3/group). The muscles were weighed for comparative analysis of their mass through the ipsilateral and contralateral ratio.

### 2.6 Sciatic nerve area and counting of the regenerated fibers

Semi‐thin transverse nerve sections were evaluated under light microscope (Leica DM5500B, Germany) camera and photographed with a high‐resolution camera (Leica DFC345 FX). Regenerated nerves total transverse area was measured with Image J software (version 1.33u, National Institutes of Health–NIH, USA; RRID:SCR_003070) and to perform the counting of the regenerated fibers, 30% of the total nerve area was photographed at 1,000× magnification (Mayhew & Sharma, 1984a, 1984b). The total number of nerve fibers was estimated from a simple rule of three, considering the total nerve area.

### 2.7 Scanning electron microscopy of the tubular prosthesis

The tubular prostheses were observed in a scanning electron microscope to evaluate their inner surface. For this, the samples were fixed with Karnovsky solution (2% glutaraldehyde, 1% paraformaldehyde, 0.1 mol/L PB, pH 7.4) and postfixed with 1% osmium tetroxide. Following postfixation, the specimens were washed in distilled water and dehydrated in increasing alcohol series. Then, samples were dried at a critical point (Balzers CTD030), metalized in a sputter coater (BAL‐TEC SCD050) with gold, and observed under a scanning electron microscope (Zeiss EVO MA15). Samples not implanted were used as controls.

### 2.8 Statistical analysis

The data are presented as mean ± *SE*, and the differences between groups were considered significant when the *p*‐value was <.05 (*), <.01 (**), and <.001 (***). Statistical analysis was performed with GraphPad Prism 5.0 software (RRID:SCR_002798). In this sense, data were subjected to ANOVA followed by Bonferroni post hoc test for parametric data or Mann–Whitney *U* test for nonparametric data.

## 3 RESULTS

### 3.1 Characterization of nanocomposites

#### 3.1.1 Fracture surfaces of the membranes

**Figure 2:**
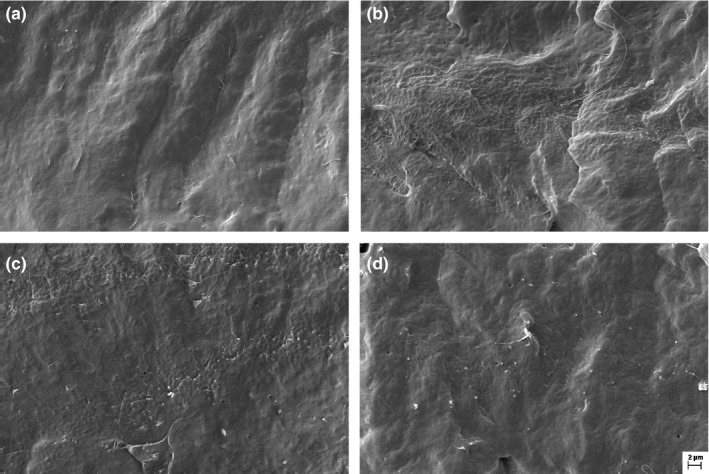
Micrographs of the tubular prosthesis inner surface before implantation: (a) PCL, (b) PCL + NPC, (c) PCL + NPG, and (d) PCL + NPC + NPG. Scale bar: 2 μm, 5,000×. PCL, poly(ε‐caprolactone); NPC, nanoparticles of carbon; NPG, nanoparticles of graphene

The images of fracture surfaces show the smooth surface of PCL‐alone membranes and the distribution of the carbon nanostructures in the polymeric matrix nanocomposites (Figure 2). Carbon nanotubes (NPC) form agglomerates, whereas the graphene oxide (NPG) and the mixture (PCL + NPC + NPG) showed a better dispersion in the polymeric matrix.

#### 3.1.2 Differential scanning calorimetry

**Figure 3:**
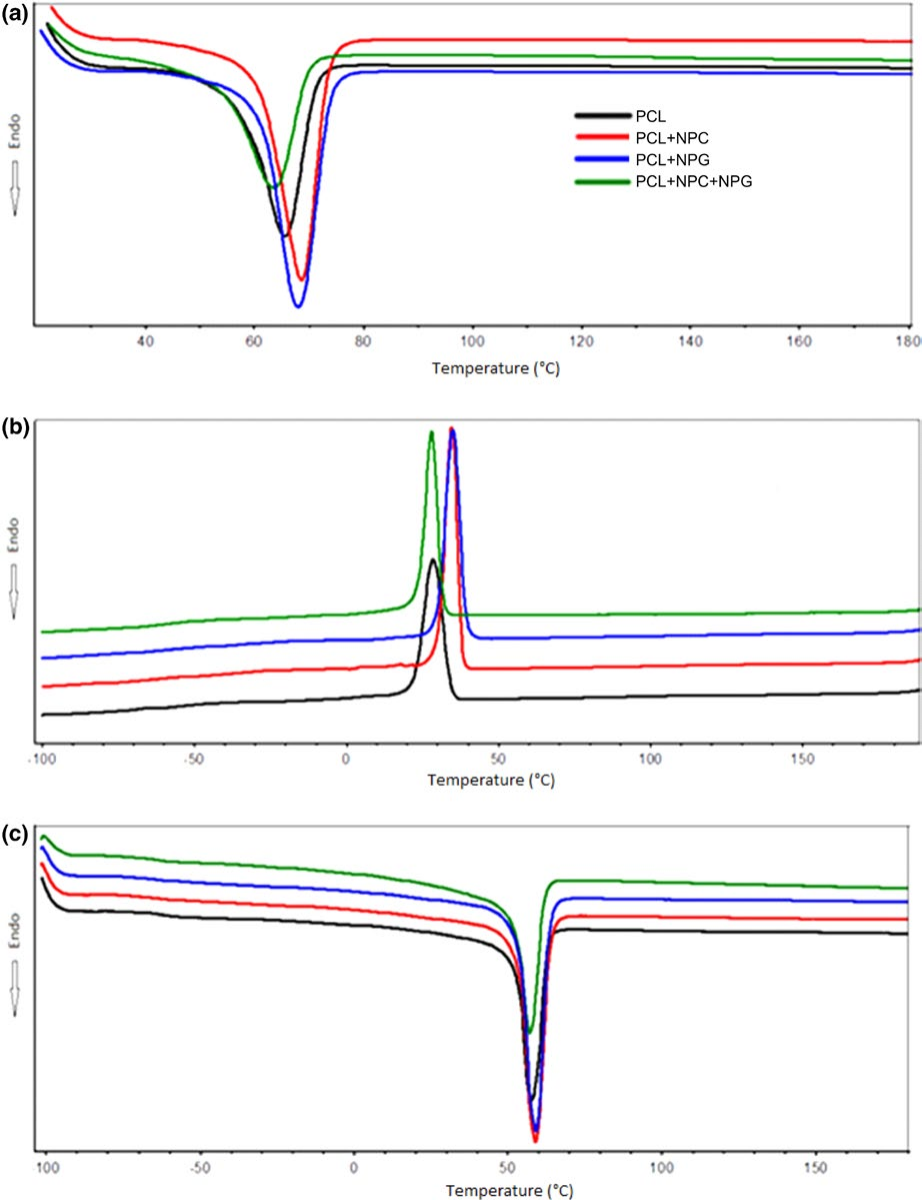
Differential scanning calorimetry (DSC) curves. (a) First heating, (b) cooling, and (c) second heating

Figure 3 shows that there were no changes in the melting temperature (*T*_m_) of the studied membranes. However, the enthalpy of melting (Δ*H*_m_) increased after the addition of NPC and NPG to the first and second heating, whereas with the addition of the mixture of PCL + NPC + NPG there was a decrease in the enthalpy of melting. As seen in Table 1, *T*_c_, Δ*H*_c_, and χ_c_ increase in pure PCL as compared to nanocomposites.

**Table 1:** Differential scanning calorimetry parameters for PCL and nanocomposites

Sample	*T*_m1_	*T*_m2_ (°C)	Δ*H*_m1_ (J/g)	Δ*H*_m2_ (J/g)	*T*_c_ (°C)	Δ*H*_c_ (J/g)	*X*_m1_ (%)	*Χ*_m2_ (%)
PCL	58	53	75	62	33	57	43	46
PCL + NPC	61	54	95	78	38	67	45	58
PCL + NPG	61	54	95	77	39	69	45	57
PCL + NPC + NPG	55	56	64	53	32	50	48	47

PCL, poly(ε‐caprolactone); NPC, nanoparticles of carbon; NPG, nanoparticles of graphene. *T*_m1_ (melting temperature), first heating; *T*_m2_ (melting temperature), second heating; Δ*H*_m1_ (enthalpy of melting), first heating; Δ*H*_m2_ (enthalpy of melting), second heating; *T*_c_ (crystallization temperature); Δ*H*_c_ (enthalpy of crystallization); χ_m1_ (degree of crystallinity), first heating; χ_m2_ (degree of crystallinity), second heating.

#### 3.1.3 Dynamic mechanical analysis

**Figure 4:**
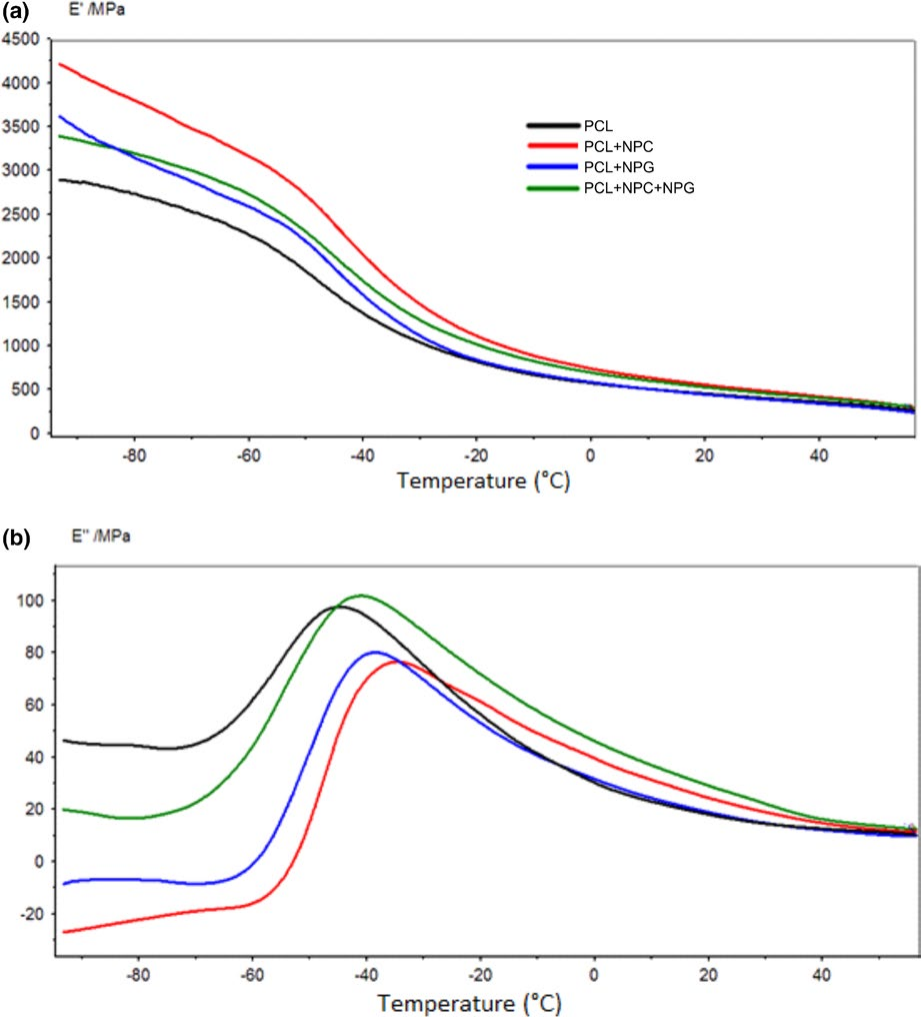
Dynamic mechanical analysis (DMA) curves. (a) E' (storage modulus) and (b) E” (loss modulus)

Figure 4 shows that addition of NPC, NPG, and their mixture caused an increased storage modulus (E'). Of note, the presence of NPC alone achieved the greatest values. The loss modulus (E”) measures the energy dissipated and the glass transition can be viewed by the peak of the loss modulus curve. The values obtained were −45°C for pure PCL, −41°C for PCL + NPC, −39°C for NPG, and −35°C for PCL + NPC + NPG.

### 3.2 Tubulization

After the tubulization of the sciatic nerve, the animals were observed during the postoperative periods, and there was no occurrence of self‐mutilation, ulcers, or joint contractures at the site of injury.

All prostheses used in this study had transparency, contributing to the correct positioning of the nerve stumps when stitched at their ends. After 8 and 12 weeks of implantation, in the four studied groups, the tubes were found in the lesion site, connecting the ends of the nerves in the same way as were implanted. Moreover, in all analyzed tubes, a nervous cable was found connecting the proximal and distal stumps.

The materials used to manufacture the prosthesis shown to be biocompatible since no infection or inflammation in the lesion site could be depicted. Moreover, a thin layer of connective tissue formed on the inner and outer surfaces of the prostheses.

### 3.3 Scanning electron microscopy of the tubular prosthesis

The inner surface of the tubular prosthesis was analyzed by scanning electron microscopy before implantation and after 8 and 12 weeks of surgery.

**Figure 5:**
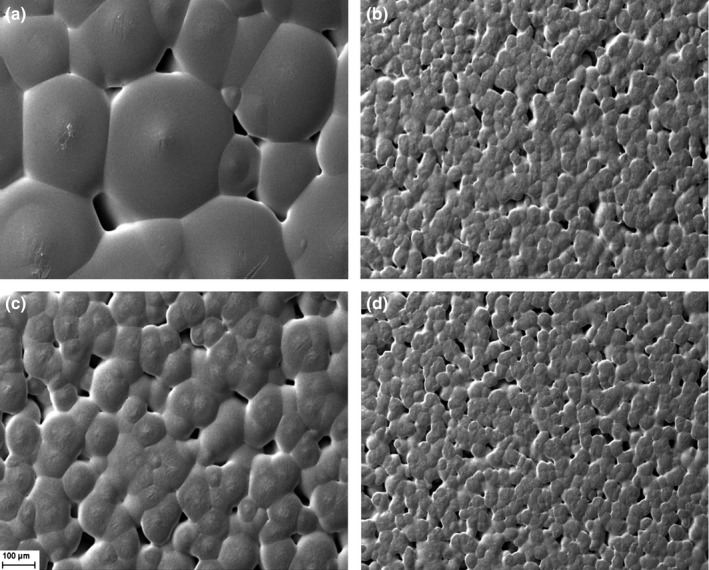
Scanning electron microscopy of the prosthesis before implantation, showing the presence of globular structures and pores. (a) PCL, (b) NPC, (c) NPG, and (d) mixture. The use of nanoparticles greatly decreased the size of such globular structures. Scale bar: 100 μm, 250×. PCL, poly(ε‐caprolactone)

Before used for the implant, all membranes presented globular structures and pores, which are formed by solvent evaporation, ranging in size and number in different studied materials here (Figure 5).

At the end of the studied periods, the globular structures and pores were rarely observed because there was deposition of extracellular matrix that overlaid the surface of the membranes. Moreover, the degradation of the material may have originated a flatter surface.

### 3.4 PCL prosthesis and association with NTC and NPG

**Figure 6:**
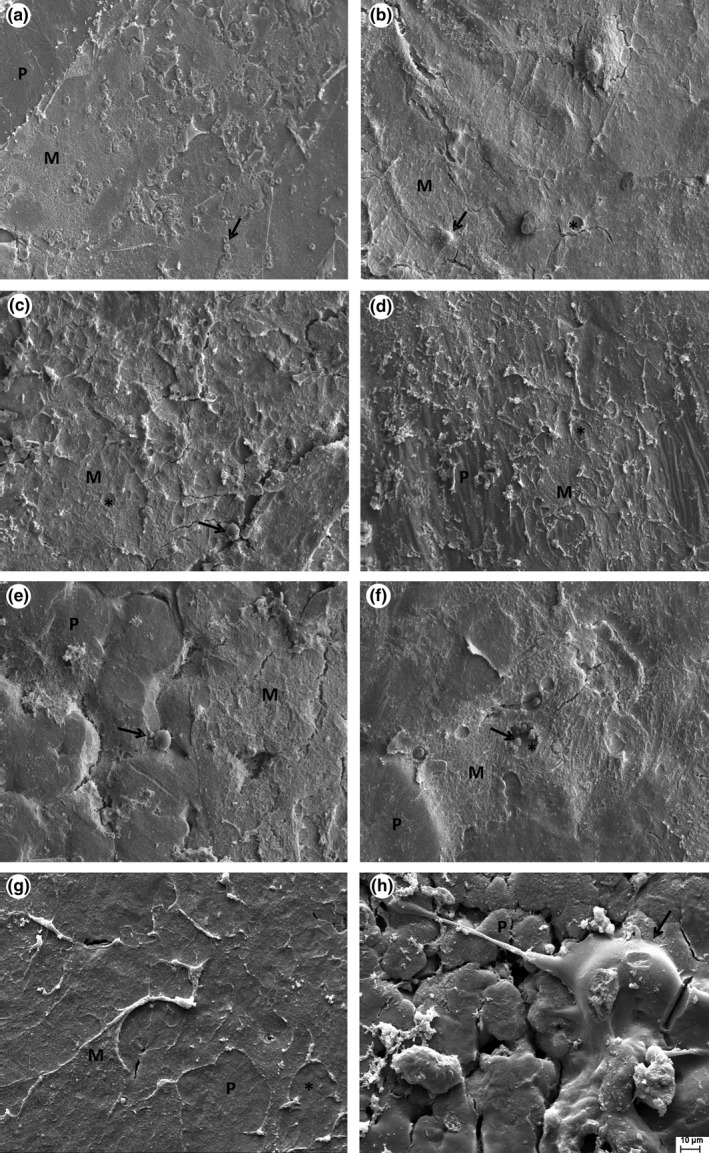
Scanning electron microscopy of the inner surface of tubular prostheses 8 and 12 weeks postimplantation. (a, b) PCL, (c, d) PCL + NPC, (e, f) PCL + NPG, and (g, h) PCL + NPC + NPG. After 12 weeks, matrix deposition was greater, but with few cells. Magnification: 800×. P, area not covered by matrix; M, area covered by matrix; arrow, cells; asterisk, marks indicative of blood vessels. Scale bar: 10 μm, 1,500×. PCL, poly(ε‐caprolactone); NPC, nanoparticles of carbon; NPG, nanoparticles of graphene

After used for the implant (8 and 12 weeks postsurgery), there was deposition of extracellular matrix in the PCL prosthesis, covering almost the entire surface (Figure 6). Moreover, PCL tubes showed many adherent red blood cells and rarely appeared another cell type. Just scanning electron microscopy (SEM) did not allow us to identify the cell types present there. Red blood cells could be recognized because they have a very distinctive shape. At 12 weeks, the deposited matrix was thicker and covered a larger area. Also, the amount of adhered cells was lower as compared to 8 weeks post implantation. In both tubes, 8 and 12 weeks postoperative, structures were seen suggesting the presence of blood vessels during tissue regeneration.

After 8 weeks of implantation, the NPC containing membranes also showed many adherent cells on its surface and, unlike the PCL‐alone group, with a smaller amount of red blood cells. Furthermore, there was a significant amount of matrix deposited on such nanocomposite membranes. After 12 weeks, the number of cells and deposited matrix were less prominent when compared to the previous period. Also, it was possible to observe structures indicating the presence of blood vessels as well as signs of degradation of the polymer.

Membranes prepared with the mixture of PCL + NTC + NPG, after 8 weeks, displayed less prominent matrix deposition as compared to 12 weeks period. Adhered cells, sending cytoplasmic projections, were compatible with the morphology of fibroblasts. Of note, polymer degradation/reabsorption appeared significantly more advanced in this group, 12 weeks post‐tubulization, presenting a large number of fissures and infiltrating cells, possibly macrophages.

### 3.5 Evaluation of nerve regeneration

#### 3.5.1 Sciatic nerve area

**Figure 7:**
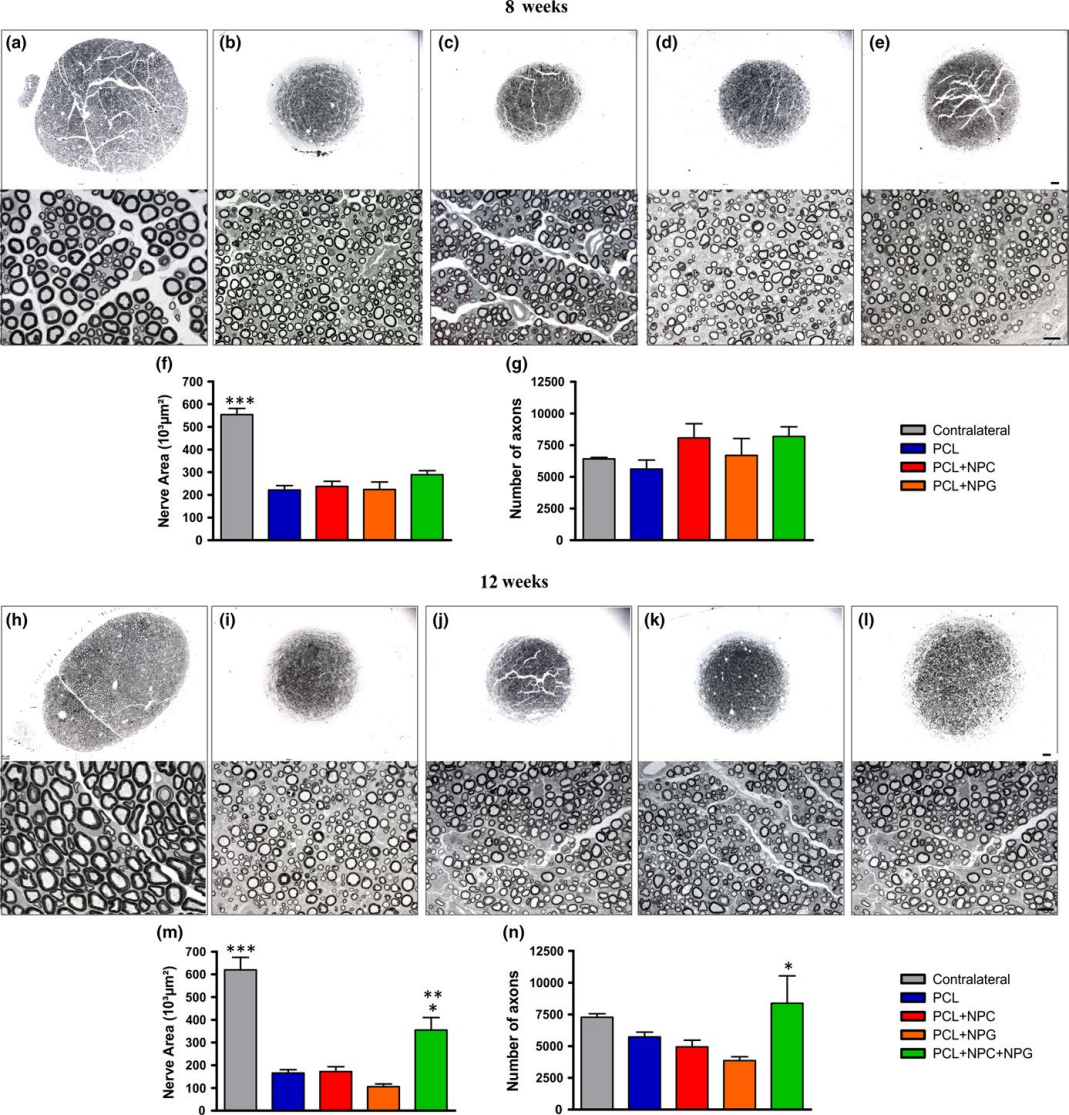
Panoramic view of regenerated nerves from the different experimental groups observed under light microscopy. Eight weeks postimplantation: (a) contralateral, (b) PCL, (c) PCL + NPC, (d) PCL + NPG, and (e) PCL + NPC + NPG. Sciatic nerve area (f) and estimated number of axons (g) found in cross sections of the regenerated nerves at tube midpoint, 8 weeks postsurgery. Overview of regenerated nerves, 12 weeks postlesion and post‐tubulization: (h) contralateral, (i) PCL, (j) PCL + NPC, (k) PCL + NPG, and (l) PCL + NPC + NPG. Sciatic nerve area (m) and estimated number of axons (n) found in cross sections of the regenerated nerves at tube midpoint, 12 weeks postsurgery. Staining: Sudan Black. Magnification: 200× and 1,000×. Scale: 50 μm and 100 μm, respectively. PCL, poly(ε‐caprolactone); NPC, nanoparticles of carbon; NPG, nanoparticles of graphene. *p*< 0.05 (*), *p*< 0.01 (**), and *p*< 0.001 (***)

The sciatic nerve area was calculated for each experimental group, at 8 (Figure 7f) and 12 weeks postsurgery (Figure 7g). At 8 weeks, only the contralateral nerve was shown to be statistically different in relation to the other groups, with *p* < .001 (contralateral: 553,800 ± 27.20; PCL: 221,600 ± 19.26; PCL + NPC: 237,200 ± 22.84; PCL + NPG: 223,600 ± 33.24; PCL + NPC + NPG: 289,000 ± 17.96; mean ± *SE*). At 12 weeks post‐tubulization, the areas of the contralateral nerves were also higher than the other groups (contralateral: 619,800 ± 54.98; PCL: 165,800 ± 14.68; PCL + NPC: 172,000 ± 21.23; PCL + NPG: 106,000 ± 11.38; PCL + NPC + NPG: 354,500 ± 55.34; mean ± *SE*,* p* < .001). However, it was observed that the mixture group also showed significant differences compared to PCL alone (*p* < .05), NPC (*p* < .05), and NPG (*p* < .01).

#### 3.5.2 Counting of regenerated fibers

After 8 and 12 weeks, the number of fibers in the regenerated sciatic nerve of each animal was also estimated. After 8 weeks, there was no statistical difference among groups (contralateral: 6,423 ± 107.1; PCL: 5,619 ± 707.5; PCL + NPC: 8,069 ± 1,119, PCL + NPG: 6,697 ± 1,329; PCL + NPC + NPG: 8,191 ± 753.1; mean ± *SE*; Figure 7g). At 12 weeks of treatment, the mixture group showed greater number of fibers, being statistically different from the other groups, with *p* < .05 (contralateral: 7,281 ± 271.0; PCL: 5,720 ± 381.2; PCL + NPC: 4,936 ± 532.8; PCL + NPG: 3,863 ± 301.3; PCL + NPC + NPG: 8,381 ± 2,158; mean ± *SE*; Figure 7n).

### 3.6 Assessment of muscles

#### 3.6.1 Ratio of the masses

**Figure 8:**
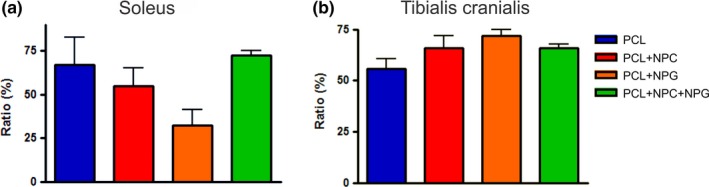
Comparison of the ratio of the muscle masses of the ipsilateral and contralateral sides. (a) Soleus and (b) tibialis cranialis

There was no statistical difference between groups (*p* > .05) when comparing the ratio of the masses of the soleus muscle at ipsilateral and contralateral sides after 12 weeks of nerve repair. The same was observed for the anterior tibialis muscle. Of note, PCL + NPC group showed the smallest ratio, indicating a trend to muscle atrophy (Figure 8).

## 4 DISCUSSION

Peripheral nerve transection interrupts the contact between spinal motoneurons as well as DRG neurons to the respective target organs, resulting in paralysis and numbness or neuropathic pain (Hoffman‐Kim, Mitchel, & Bellamkonda, 2010). Thus, reconnection of proximal and distal stumps is necessary to allow regrowth of axons from the proximal stump toward the periphery. To date, autografting is the golden standard approach if end‐to‐end neurorrhaphy is not possible. Nevertheless, with the advent of nanotechnology, new possibilities have been proposed, so that alternatives to autografting may restore function appropriately, avoiding the unwanted effects of nerve transfer/grafting.

Nanocomposites of bioresorbable polymers associated with nanoparticles of carbon can provide mechanical support and even stimulate axonal growth by facilitating cell migration (Mattson et al., 2000). Herein, we show that the combination of PCL + NPC + NPG produces homogeneous membranes that can be rolled into tubular prostheses and used for tubulization of sciatic nerve post‐transection (neurotomy). In a previous study, we have already demonstrated that PCL alone allows proper nerve regrowth and successful reinnervation of hind limb musculature (Pierucci et al., 2008). By using the solvent evaporation approach, thin and resistant tubes are produced, and the combination with NPC + NPG improved significantly chemical and mechanical properties of the composite.

Transparency and flexibility were improved in PCL + NPC + NPG tubes, possibly by the formation of smaller granular polymer structures as seen by MEV. This is consistent with the concept that a nerve conduit must be flexible and yet resistant to mechanical forces, avoiding the collapse of its walls (de Ruiter, Malessy, Yaszemski, Windebank, & Spinner, 2009; Sun & Downes, 2009). Biocompatibility is another parameter of pivotal importance. Herein we demonstrate that regeneration occurred in all groups. However, the nanocomposites showed enhanced interaction with the microenvironment of the regenerating nerve. Such interaction allowed the formation of blood vessels and, already at 12 weeks, some putative phagocytes were present within composite fissures, indicating faster degradation rate, as compared to PCL alone. The better environment reorganization may also be due to the enhanced dispersion of nanoparticles in the PCL polymer when NPC and NPG were combined. As described previously, NPC forms aggregates, resulting in nonhomogeneous preparations. On the contrary, graphene sheets (NPG) provide improved dispersion properties (Sun et al., 2013). In turn, the combination of NPC + NPG produced the best results, taking advantage of positive characteristics of both nanoparticles.

Cell proliferation and migration to the inner tube space is of fundamental importance for a successful regenerative process (Dubey, Letourneau, & Tranquillo, 1999; Geuna et al., 2009). It is known that such space is quickly filled with plasma exudate composed mostly of a fibrin network and platelets. It has been recently shown that blood vessel formation and polarized growth precedes Schwann cell migration, which in turn produce the so‐called Bands of Büngner (Cattin et al., 2015). Such cell cords guide the growing axons toward the distal stump of the nerve.

Importantly, implanted tubular prostheses are enclosed by connective tissue both at the outer and inner wall surfaces. In this sense, the use of hydrophilic biomaterials may result in thick encapsulation and, in turn, compression of the regrowing nerve. This can be avoided by using PCL, which is hydrophobic (Subramanian, Krishnan, & Sethuraman, 2009). Nevertheless, the balance provided by mixing PCL with NPC and NPG results in a biofunctional nanocomposite that allowed only a thin layer of tissue covering, what possibly facilitated the regenerative process. In this regard, nanostructures of carbon, due to their negative charge, may facilitate the initial steps of cell adhesion and proliferation (Bergethon, Trinkaus‐Randall, & Franzblau, 1989; Lee, Cuddihy, & Kotov, 2008; Oliveira et al., 2014; Zhu et al., 2010). As a result, significant larger nerve bridges were found within PCL + NPC + NPG tubes, which presented greater number of myelinated axons.

One key point for the success of nerve tubulization by using bioresorbable polymers is the composite degradation rate (Moroder et al., 2011). As mentioned earlier, PCL alone presented larger globular structures as compared to the nanocomposites. In this sense, the increased surface of contact possibly facilitated hydrolysis and the initial steps of degradation, generating fissures and cavities already at 12 weeks postsurgery. Nonetheless, at that stage the nerve cable is well formed and stable, so that reabsorption of the composite should not interfere in the functional motor/sensory outcome. The process of hydrolysis, however, if too fast, may generate oxidative stress and a local decrease in pH (de Ruiter et al., 2009). Based on the fact that muscle mass was preserved in all tubulized groups, no harmful effect of the prosthesis degradation should have affected the newly formed nerve (Dow et al., 2004; Yu et al., 2011).

In conclusion, the present work indicates that combining PCL with nanoparticles of carbon and graphene is a promising approach, since such association improves chemical/mechanical aspects of the composite, even accelerating its degradation rate. Also, biocompatibility is enhanced, as NPC and NPG are negatively charged, facilitating cell migration into the tube. Overall, nerve regeneration was more prominent when PCL + NPC + NPG was used, generating thicker nerve cables, containing increased number of myelinated axons.

## CONFLICT OF INTEREST

None declared.
